# Antimicrobial Wound Dressings based on Bacterial Cellulose and Independently Loaded with Nutmeg and Fir Needle Essential Oils

**DOI:** 10.3390/polym15173629

**Published:** 2023-09-01

**Authors:** Georgiana-Madalina Lemnaru (Popa), Ludmila Motelica, Roxana Doina Trusca, Cornelia Ioana Ilie, Alexa-Maria Croitoru, Denisa Ficai, Ovidiu Oprea, Anicuta Stoica-Guzun, Anton Ficai, Lia-Mara Ditu, Bianca-Maria Tihăuan

**Affiliations:** 1 National Centre for Micro and Nanomaterials and National Centre for Food Safety, University Politehnica of Bucharest, Splaiul Independentei 313, 060042 Bucharest, Romania; 2 Department of Science and Engineering of Oxide Materials and Nanomaterials, Faculty of Chemical Engineering and Biotechnologies, University Politehnica of Bucharest, Gheorghe Polizu 1-7, 060042 Bucharest, Romania; 3 Department of Inorganic Chemistry, Physical Chemistry and Electrochemistry, Faculty of Chemical Engineering and Biotechnologies, University Politehnica of Bucharest, Gheorghe Polizu 1-7, 060042 Bucharest, Romania; 4 Faculty of Biology, University of Bucharest, 1-3 Aleea Portocalelor, 060101 Bucharest, Romania; 5 Academy of Romanian Scientists, 3 Ilfov Street, 050045 Bucharest, Romania; 6 Department of Chemical and Biochemical Engineering, Faculty of Chemical Engineering and Biotechnologies, University Politehnica of Bucharest, Gheorghe Polizu 1-7, 060042 Bucharest, Romania; 7 Research Institute of the University of Bucharest—ICUB, 91-95 Spl. Independentei, 50567 Bucharest, Romania; 8 Research & Development for Advanced Biotechnologies and Medical Devices, SC Sanimed International Impex SRL, 087040 Călugăreni, Romania

**Keywords:** bacterial cellulose, nutmeg essential oil, fir needle essential oil, antimicrobial, treatment of skin, wound dressing

## Abstract

The aim of the present study was to obtain antimicrobial dressings from bacterial cellulose loaded with nutmeg and of fir needle essential oils. The attractive properties of BC, such as biocompatibility, good physicochemical and mechanical stability, and high water absorption, led to the choice of this material to be used as a support. Essential oils have been added to provide antimicrobial properties to these dressings. The results confirmed the presence of oils in the structure of the bacterial cellulose membrane and the ability of the materials to inhibit the adhesion of *Staphylococcus aureus* and *Escherichia coli*. By performing antibacterial tests on membranes loaded with fir needle essential oil, we demonstrated the ability of these membranes to inhibit bacterial adhesion to the substrate. The samples loaded with nutmeg essential oil exhibited the ability to inhibit the adhesion of bacteria to the surface of the materials, with the 5% sample showing a significant decrease. The binding of essential oils to the membrane was confirmed by thermal analysis and infrared characterization.

## 1. Introduction

Skin and subcutaneous diseases represent a real public health problem worldwide, affecting millions of people. Their incidence increases with age, comorbidities such as diabetes, and with a sedentary lifestyle. The risk of chronic wound infections increases with delayed healing, so it is necessary to provide regular care to prevent or control the infection of these wounds until they are completely healed. Chronic therapies are very costly and depend on the efficiency of developing suitable materials that can mimic the extracellular matrix and provide effective antimicrobial compounds. In the case of infection, critical colonization or localized infection, without appropriate treatment, can lead to the need for surgery to prevent systemic infection or even death of the patient [1].

Thus, materials suitable for these medical problems should provide a moist environment to reduce the risk of scarring while promoting epithelialization and the migration of cells to the wound. Other properties required for these types of materials are mechanical stability during application, under a certain amount of pressure and tension, and the ability to act as a barrier against external threats such as microbes, foreign bodies, or tissue damage [2,3]. Although conventional wound dressings contribute to wound healing, they are not as effective as desired, their practical use being hindered due to challenges in maintaining adequate moisture of the wound or their tendencies to adhere to the tissue. Even though conventional dressings are frequently employed in clinical practice, as a cost-effective option, it is important to note that these types of dressings primarily provide physical protection and offer minimal advantages in terms of promoting wound healing and preventing infections [4]. Therefore, new solutions are needed to create a medical device that meets all of the above requirements. Combining a simple dressing with an active ingredient is one of solutions [5,6]. Moreover, a wide range of materials are now used for wound dressings, such as synthetic or natural polymers [7]. Each category of material exhibits advantages and disadvantages. However, natural polymers are most often chosen, especially because they show better biocompatibility, non-toxicity, and biodegradability compared with synthetic polymers [8]. A disadvantage of natural polymers, with the exception of chitosan, is their lack of antimicrobial activity. So far, in order to obtain bioactive dressings, it has been necessary to incorporate an antimicrobial agent into the polymer matrix. As a matter of fact, combining macromolecules such as polysaccharides, proteins, and lipids with various active substances can yield useful properties that are absent in individual constituents [9]. The use of saccharides such as cyclodextrins offers a good alternative for their use as host molecules, thus improving the stability and bioavailability of various active substances. For example, Fan et al. [10] observed an improved antimicrobial effect of gallic acid on a strain of *E. coli.* Moreover, the encapsulation of various active substances has gained momentum, not only in the medical field, but also in crop treatment, for example, by incorporating pesticides into beta-cyclodextrin-derived nanofibers [11].

Bacterial cellulose (BC), which is a natural biopolymer, has been found to meet many of the criteria for use in wound healing [12], as well as for other medical applications [13].

It is produced via aerobic fermentation by strains of the Gram-negative bacteria *Komagateibacter* (formerly known as *Glucanoacetobacter* and *Acetobacter*), being the most productive type [14]. The obtained pellicles are further purified, usually by an alkaline treatment, which is considered as a standard procedure for removing bacteria and culture medium from BC gel membranes [15]. This treatment can ensure a total loss of the viability of the bacterial cells. Biological impurities from the bacteria or fermentation medium could remain attached to the BC network, but in very small quantities. The NaOH treatment is considered to be able to eradicate bacteria by cell lysis. Nevertheless, concentrations of NaOH higher than 5% are not recommended, as this could change the crystalline structure of BC from cellulose I to cellulose II, leading to the disruption of several inter- and intra-molecular hydrogen bonds inherent in cellulose, which further decreases the mechanical properties of the pellicle [16,17]. It can also be used in tissue engineering products for wound care and the regeneration of diseased skin or organs, being an extremely versatile biomaterial [18]. The physicochemical properties of the material, such as high mechanical strength, biocompatibility, a three-dimensional fibrillary nanostructure, as well as high hydrophilicity, exudate absorption, and moisture retention, make BC ideal for a wound dressing. High mechanical strength in the wet state, substantial permeability to liquids and gases, and low skin irritation indicated that this BC membrane could be used as artificial skin for temporary wound coverage [19]. However, there is a drawback, namely its lack of antimicrobial properties. Previous studies have tried to overcome this disadvantage by incorporating or grafting active substances, such as antibiotics, nanoparticles, essential oils, etc., into the BC structure [20,21,22].

Oliveira et al. (2019) [20] conducted studies on BC membranes used as innovative dressings for skin wounds, incorporating propolis microemulsions; these dressings showed remarkable antibacterial activity in vitro and a wide range of treatments for infected wounds.

The emergence of drug-resistant microorganisms, such as methicillin-resistant *Staphylococcus aureus* (MRSA) and extended-spectrum beta-lactamases (ESBLs) *Escherichia coli,* due to the uncontrolled use of conventional antibiotics and synthetic antimicrobials (especially when combined with other pollutants such as heavy metals, pesticides, etc.) represent an increasing threat worldwide. Therefore, it is necessary to find alternatives and effective ways to treat such infections. Essential oils (EO), with their multi-target components alone, or in association with other antimicrobials agents (especially nanoparticles), are promising candidates able to reduce the risk of developing microbial resistance [23].

EOs are a mixture of bioactive components such as terpenes, terpenoids, and phenolic compounds that act synergistically to protect plants from environmental hazards. The antimicrobial properties of essential oils are the results of these bioactive compounds. The mechanism of action is based on the irreversible destruction of the bacterial cell wall and membrane. This mechanism has led to the use of essential oils in the treatment of infections since ancient times [24]. In a recent study, Czlonka et al. (2020) [25] reported the antimicrobial activity of nutmeg oil against *Staphylococcus aureus* and *Escherichia coli*, with higher concentrations of this oil showing greater inhibition for these bacteria. Compounds responsible for the antimicrobial activity of this essential oil, also reported in other studies, are trimyristin, myristic acid, α-pinene, β-pinene, p-cymene, β-caryophyllene, and carvacrol [25,26,27]. Nutmeg oil also has excellent radical scavenging properties, reduces metal ions, and inhibits lipid oxidation [28]. Nutmeg oil has several uses that have been substantiated by clinical trials. It exhibits aphrodisiac effects, antimicrobial and antifungal effects, antioxidant and analgesic effects, as well as antidepressant and antitumor activity [29].

Another essential oil that has been used in traditional medicine to prevent infections is fir needle essential oil. Thanks to its high concentration of antiseptic organic compounds that stimulate the immune system and prevent dangerous infections, fir needle essential oil can be a powerful tool in keeping the human body healthy, inside and out. Some of the most common benefits of fir needle essential oil include: natural antiseptic, antibacterial and antimicrobial properties, infection prevention, detoxification, improved respiratory function, etc. [30].

Zubaid-ul-khazir et al. (2021) [30] investigated the properties of the essential needle oil of *Abies pindrow* and have demonstrated its antibacterial activity against various bacterial strains. Hofmann et al. (2020) [31] studied different types of fir cones for their health benefits, including anti-inflammatory, antioxidant, antimicrobial, and analgesic effects. Fir cone extracts could aid in the healing process of the common cold, urinary tract and kidney infections, dermatological disorders, bronchitis, pneumonia, and various other diseases. Polyphenolic compounds have been shown to be the most potent antioxidant and ascorbic radical scavengers in these extracts.

The aim of this study is to develop and characterize a new antibacterial wound dressing by incorporating into the BC structure two essential oils: nutmeg and fir needle. The loading of the essential oils was studied, and the antibacterial properties of the new wound dressings were assessed.

## 2. Materials and Methods

### 2.1. Materials

The two essential oils used in this study, nutmeg and fir needle essential oil, were obtained from Roth (Roth, Germany). In order to produce the desired materials, emulsions of these oils had to be obtained using Tween 80, produced by Roth (Germany), as well as ethanol and glycerin, produced by Sigma-Aldrich (Darmstadt, Germany). Deionized water was used during the assays. All these reagents were of reactive grade and were used without additional purification. The antimicrobial assessments were performed utilizing Nutrient Broth No 2 and Agar (microbiological grade) Sigma-Aldrich (Germany).

### 2.2. Methods

#### 2.2.1. Bacterial Cellulose (BC)

The BC membranes were obtained after 7 days in static culture using a Hestrin-Schramm medium containing 3% fructose, as used in one of our previous studies [32]. The *Acetobacter* sp. strain used in this study was isolated from traditionally fermented vinegar in the Microbiology Laboratory of the Chemical and Biochemical Engineering Department of the University Politehnica of Bucharest. The obtained gel-like pellicles were purified by boiling in 0.1 M aqueous solution of NaOH for 1 h and rinsing several times with deionized water until the pH of the washing solution became neutral.

#### 2.2.2. Synthesis of the BC Membranes Loaded with Essential Oils

**Figure 1:**
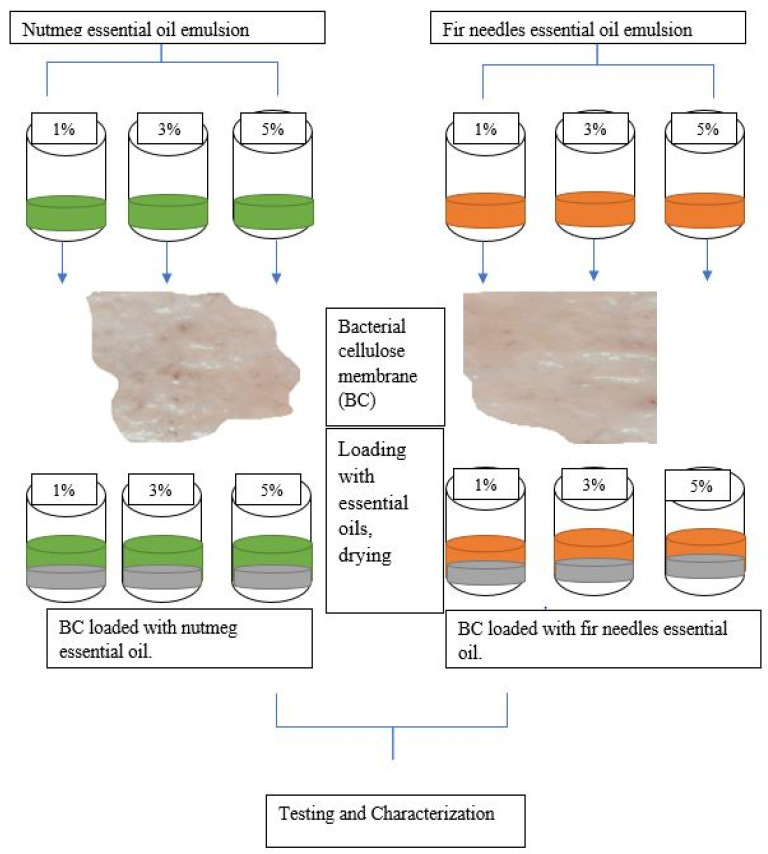
The synthesis flow of loading BC membranes with nutmeg and fir needle essential oils.

The first problem to be solved results from the high water content of BC membranes (up to 99%) and the lack of solubility of the essential oils in water. For this reason, the emulsions of essential oils were obtained as a first step. Tween 80 was used as an emulsifier and glycerin as a plasticizer. Three different concentrations of essential oil emulsions in ethanol were tested: 1%, 3%, and 5% (*v/v*). The BC membranes were immersed in the emulsions for 24 h and then held in an oven at a temperature of 37 °C for 48 h. The technological flow of loading BC membranes with nutmeg and fir needle essential oils is presented in Figure 1.

The loading of the two oils into the structure of the BC membrane was carried out using their emulsions, prepared at the concentrations of 1, 3, and 5% in ethanol, and 2% of Tween 80. The role of the surfactant is to reduce the surface tension of the liquids, thus facilitating dispersion. At the same time, glycerin is responsible for providing a more flexible structure to this dressing membrane used for skin wounds.

#### 2.2.3. FTIR Analysis

The synthesized samples were characterized by FTIR using a Nicolet iS50 FT-IR (Nicolet, MA, USA) spectrometer equipped with a DTGS detector, providing information with high sensitivity over the range of 4000 and 400 cm^−1^ at a resolution of 4 cm^−1^. All spectra were achieved by co-adding 32 scans, with a scanning time of 47 s.

#### 2.2.4. SEM Analysis

The SEM-EDS characterization was performed using a QUANTA INSPECT F50, FEI Company, Eindhoven, The Netherlands, scanning electron microscope equipped with field emission gun electron-FEG (field emission gun) with 1.2 nm resolution and an energy dispersive X-ray spectrometer (EDS) with an MnK resolution of 133 eV. Prior to analysis, the samples were sputter coated with silver.

#### 2.2.5. Thermal Analysis

TG-DSC thermal analysis of the samples was performed with an STA TG/DSC Netzsch Netzsch Jupiter 449 F3 equipment (Selb, Germany). Approximately 10 mg of each sample was placed in an open crucible made of alumina and heated with 10 K·min^−1^ from room temperature up to 900 °C, under the flow of 50 mL min^−1^ of dried air. An empty alumina crucible was used as the reference. The evolved gases were analyzed with an FTIR Tensor 27 from Bruker (Bruker Co., Ettlingen, Germany), equipped with a thermostated gas cell.

#### 2.2.6. Water Absorption

The water absorption capacity of the BC samples loaded with fir needle and nutmeg essential oils was evaluated by determining the change in sample weight during adsorption in distilled water for 72 h. The measurements were performed at different time intervals: 5, 10, 20, 30, 45, and 60 min, as well as after 2, 4, 6, 8, 12, 24, 48, and 72 h. The assay was performed in three independent experiments.

#### 2.2.7. Gas Chromatographic—Mass Spectrometric Evaluation

An Agilent Technologies 7890B GC system, Santa Clara, CA, USA, equipped with a Q-TOF 7200 mass spectrometer and an HP-5MS Ultra Inert analytical column (30 m long × 0.25 µm internal diameter × 0.25 μm film thickness), was used for analysis. The operating conditions were pulsed splitless injection mode, 280 °C injectors, and 250 °C interface temperature, 0.3 min sampling time, and helium gas as a carrier, with a flow rate 1 mL/min, 17 mL/min total flow rate, 1 mL/min column flow, 0.8 mL/min purge flow, and injection volume of 0.8 μL. The temperature was programmed from an initial value of 45 °C, ramped to 200 °C at 4 °C/min, and increased to 280 °C at 7 °C/min for 10 min, and the total run time was 63 min. The Q-TOF 7200 mass spectrometer was operated in electron ionization (EI) mode, 70 eV, with a mass range evaluation between 41 and 850 amu.

The bacterial cellulose samples loaded with essential oils were extracted using methanol as a solvent. Each sample (0.4 g) was weighed into a 15 mL centrifuge tube and extracted with 10 mL of methanol by mixing the samples for 5 min. The obtained extracts were diluted 1:1 (*v/v*) in methanol and analyzed by GC/MS Q-TOF. The essential oils were diluted 1:1000 (*v/v*) in methanol.

Chromatograms and spectra were recorded and processed using MassHunter WorkStation software B.05.00, and the spectra library used was NIST 2005 v.2.0 D). In order to avoid the possibility of false peaks from the extraction solvent, the blank sample was run by GC-MS, with solvent (methanol) only, under the same working conditions. The blank samples showed no peaks in the chromatogram, indicating the purity of the solvent used.

#### 2.2.8. Biocompatibility

Biocompatibility evaluation of the coded samples: BC-Control, BC-Nutmeg 1%, BC-Nutmeg 3%, BC-Nutmeg 5%, BC-Fir needle 1%, BC-Fir needle 3%, BC-Fir needle 5%, Nutmeg essential oil, Fir needle essential oil, was performed using the MTT colorimetric assay.

Assessment of the cellular metabolism of the samples was performed on NCTC L929 murine fibroblasts. Before testing, cells were cultivated in DMEM medium supplemented with 2 mM Glutamine (Sigma-Aldrich, St. Louis, MO, USA), 10% heat-inactivated Fetal Bovine Serum (FBS, Sigma-Aldrich) and 1% Pen/Strep (penicillin/streptomycin solution, 50 µg/mL—Sigma-Aldrich) for 24 h at 37 °C, 95% humidity, with 5% CO_2_. After 24 h, cells were washed with PBS (Phosphate Buffered Solution—Sigma-Aldrich), harvested using trypsin (Sigma-Aldrich), and counted using Trypan Blue (Sigma-Aldrich) and a hemocytometer. The seeding density for the MTT assays was optimized at 4 × 10^5^.

In a 96-well cell culture plate, cells were seeded at a density of 4 × 10^5^, treated with samples and controls, and incubated for 24 and 48 h at 37 °C, 95% humidity, with 5% CO_2_. After 24 and 48 h of exposure to the tested compounds, the cells were incubated for 4 h with MTT reagent (Roche) at 37 °C, 95% humidity, with 5% CO_2_. After incubation, the purple formazan crystals formed were dissolved with MTT solvent (Roche) for 15 min at room temperature. Absorbance was measured using a spectrophotometric microplate reader (Multiskan FC Thermo Scientific, Waltham, MA, USA) at OD = 570 nm.

#### 2.2.9. Antibacterial Evaluation

The antibacterial properties were evaluated against standard strains *S. aureus* ATCC 25923 and *E. coli* ATCC 25922 by quantitatively determining the capacity of bacteria to adhere to the surface of BC-oil samples using the method described in the previous study [32] and according to Clinical Laboratory Standard Institute standard M100. The nutmeg and fir needle essential oils and BC were used as controls. The liquid media, with bacterial cell suspensions, were used as the cell growth control. Also, the negative control (liquid media) was used to confirm the sterility of the experiments.

#### 2.2.10. Statistical Analysis

Assays were performed in three independent experiments, and the data results were statistically analyzed with GraphPad Prism 9.5 (GraphPad Software, San Diego, CA, USA). The differences between groups were compared using analysis of variance (ANOVA) and Dunnett’s multiple comparisons tests; a *p*-value < 0.05 was considered statistically significant.

## 3. Results

BC membranes loaded with the two essential oils were characterized by various physicochemical methods to determine their antimicrobial properties, biocompatibility, microstructure, stability, degree of water absorption, and the degree of loading of these membranes with essential oils.

### 3.1. Scanning Electron Microscopy (SEM)

**Figure 2:**
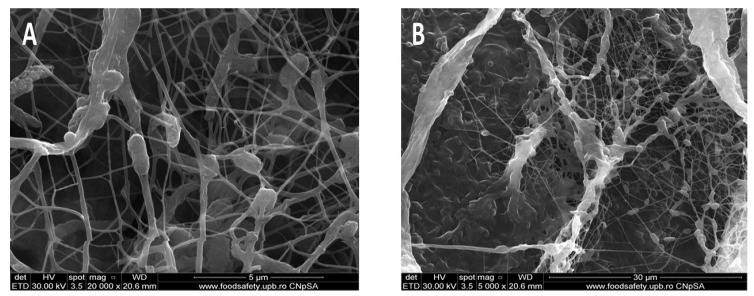

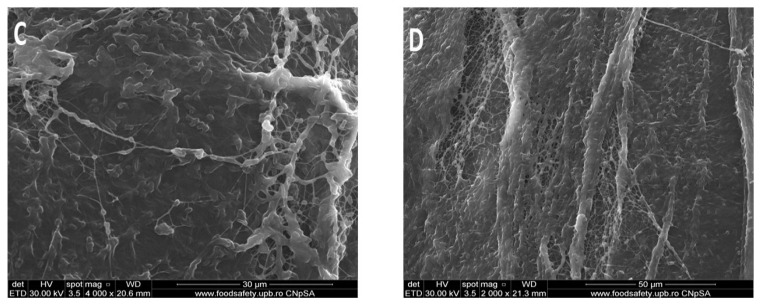
SEM images for BC-Control sample at different magnitudes (**B**) 5000×, (**D**) 2000×, and (**A**) 20,000×, (**C**) 4000×.

The pristine BC membrane was analyzed by scanning electron microscopy to determine its microstructure. Figure 2 shows a porous three-dimensional structure, which is confirmed by the literature [33,34,35,36,37,38]. As these membranes are to be used as dressings for skin wounds, the porous structure contributes to a good absorption of exudate released from the wound and the release of the essential oils at the wound surface, while protecting against pathogens and ensuring a faster tissue regeneration [19,36,39]. As shown in Figure 2A, the pores are less than 2–3 µm, but even in the case of viruses, the membrane/matrix acts as an efficient sieve and limits the penetration of these pathogens to the wound. If these dressings contain antimicrobial agents, the penetration time would be longer than that required to destabilize the pathogens, and thus the infection risk would decrease considerably.

### 3.2. FT-IR Analysis

FT-IR spectroscopic analyses were performed to verify that the BC retained its structure during the loading processes and that the essential oils were absorbed into the BC structure and on its surface.

**Figure 3:**
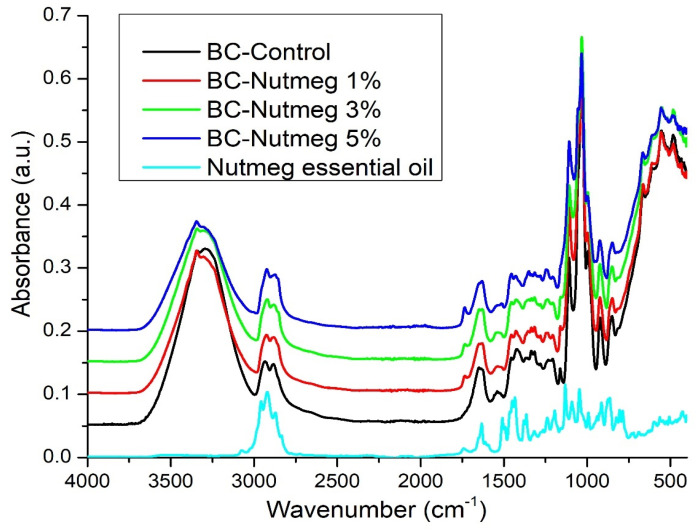
FTIR spectra of BC-Control, BC-Nutmeg 1%, BC-Nutmeg 3%, BC-Nutmeg 5%, and Nutmeg essential oil.

Figure 3 shows the control spectra of BC, BC-Nutmeg 1%, BC-Nutmeg 3%, and BC-Nutmeg 5%, along with the control of nutmeg essential oil. By analyzing the FT-IR spectrum of BC, its characteristic chemical bonds can be observed. It can be seen that at 3292 cm^−1^, there is a stretching vibration of the OH groups, and at 2933 cm^−1^, there is a stretching vibration of the CH_2_ and CH groups. The HOH binding vibration of the crystallization water is identified at 1647 cm^−1^ and 1335 cm^−1^, and the OH deformation vibration can be observed. The peak at 1206 cm^−1^ is attributed to the CH deformation vibration. The asymmetric stretching vibrations of C-C are identified at 1161 cm^−1^ [40,41,42]. The shifts of the essential oil bands on the one hand, as well as the shift of the characteristic BC bands, on the other hand, are indicators of the interaction between EON and BC [38]. The interaction of nutmeg essential oil with the BC support can be seen by the shift of the characteristic band of nutmeg essential oil from 1741 cm^−1^ to 1734–1733 cm^−1^. Another interaction of the donor materials occurs due to the shift in the oil band from 2920 cm^−1^ to 2922–2923 cm^−1^. The maximum peak at 1032 cm^−1^ of the blank BC sample undergoes a shift to 1033 cm^−1^. The same is observed in the samples loaded with fir needle essential oil.

The FTIR spectrum of nutmeg oil with bands at 1632, 1508, 1449, 1433, 1131, 1091, 1046, and 806 cm^−1^ confirms the presence of myristin in its composition [43]. Characteristic peaks located at 2957 cm^−1^ (vibration -OH) and 2920 cm^−1^ (vibration -CH_2_) correspond to the presence of cellulose, hemicellulose, and lignin. The band located at 1741 cm^−1^ corresponds to the carbonyl vibration (C=O). The peak centered at 1632 cm^−1^ indicates the presence of a symmetrical aromatic ring (aromatic C=C). The presence of flavonoids can be observed based on their characteristic peaks in the range of 1480–1440 cm^−1^. Phenol-characteristic bands can be found at 1170–1110 cm^−1^ (C–OH tensile vibration) [25,43].

It is important to note that there are interactions between the BC support and the nutmeg essential oil, as evidenced by the shift in some specific bands.

**Figure 4:**
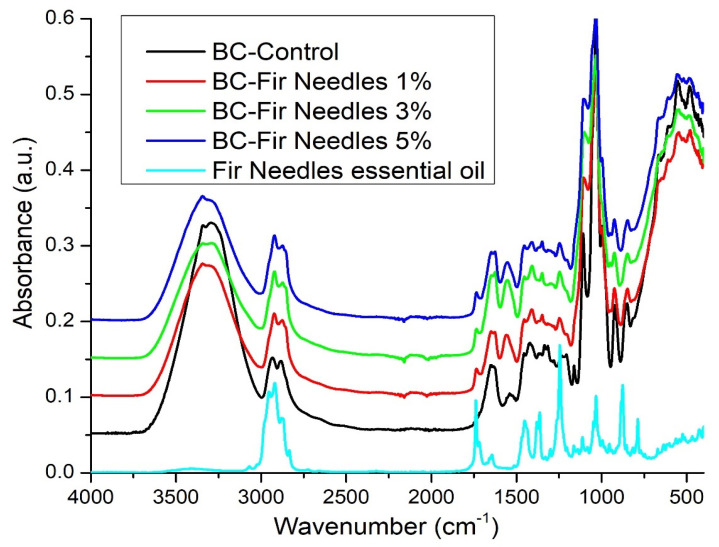
FTIR spectra of BC-Control, BC-Fir needle 1%, BC-Fir needle 3%, BC-Fir needle 5%, and Fir needle essential oil.

The FTIR spectrum of fir needles highlights some distinctive peaks, as follows. The O–H band associated with the stretching vibrations is centered at 3401 cm^−1^. The peaks between 1112 and 635 cm^−1^ are due to the C–C and C–N stretching vibrations. The peak at 1363 cm^−1^ indicates the C–N stretching vibration within the amine groups or the C–O vibration of the carboxylic acid groups [44] (Figure 4).

A large band in the 3000–3600 cm^−1^ region overlaps the vibration range of the intramolecular hydrogen bonds involving hydroxyl groups in cellulose (νOH). These bands have a maxima around 3394 cm^−1^ for young cones, 3346 cm^−1^ for elder cones, and ~ 3350 cm^−1^ for bark and needles. The absorption bands at 2919 and 2878 cm^−1^ correspond to the asymmetric and symmetric νCH_2_ [45,46].

The tensile vibration of the C=O double bond can be observed at 1738 cm^−1^. The band specific to fir needle essential oil is identified at 1738 cm^−1^ and shifts to 1734 cm^−1^ as a result of the interaction with the bacterial cellulose support. In the case of the control sample BC-Control, the maximum peak is recorded at 1032 cm^−1^ and shifts to 1033–1034 cm^−1^ in the case of the samples loaded with fir needle essential oil, due to the interactions between the support and the essential oil. Given the fact there is a low content of fir needle essential oil, the shift is moderate, but significant [47]. The band at 2878 cm^−1^, specific to fir needle essential oil, shifts to 2873–2874 cm^−1^ for BC and essential oil samples, and this shift is due to the interaction between the support and the essential oil. Comparing the recorded shifts in the two cases, it seems that BC interacts more with the nutmeg than with the fir essential oil (the shift is ~8–9 cm^−1^ in the case of nutmeg and only ~4 cm^−1^ in the case of fir).

### 3.3. Thermal Analysis

**Figure 5:**
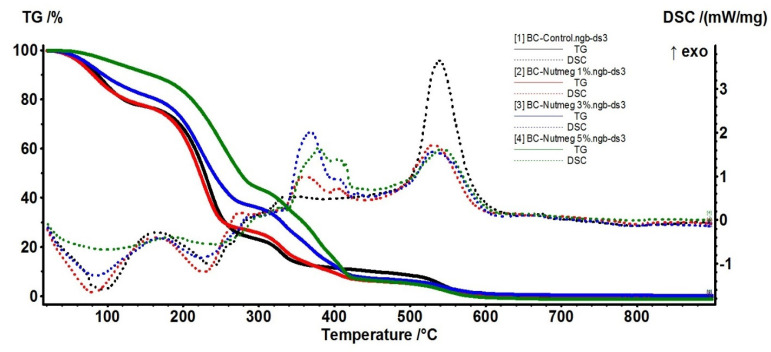
TG-DSC curves for BC-Control, BC-Nutmeg 1%, BC-Nutmeg 3%, BC-Nutmeg 5%, and Nutmeg essential oil.

The samples were further characterized by thermal analysis in order to assess the thermal stability of the samples, the composition, and the stabilization effect of the BC matrix, but also the release behavior of the EO from these membranes. The BC-Control sample presents several distinct mass loss steps. In the first step, up to 160 °C, the water molecules, physically adsorbed by the cellulosic structure, are eliminated. This accounts for 23.51% of the initial mass. An endothermic effect, with a minimum of 94.6 °C, accompanies the process. The hydrogen bonds between water and the cellulose chains are responsible for the higher temperature needed to eliminate the water. In the second process, between 160–300 °C, the cellulosic fibers start to decompose. This is the main mass loss step, with a value of 53.51%. An endothermic effect with a minimum of 240.1 °C accompanies this process. The FTIR spectra recorded for the evolved gases indicate the presence of principally water and carbon dioxide molecules, but also some traces of carbon monoxide and hydrocarbon fragments. The carbonaceous residue burns off at a temperature interval of 300–440 °C. The process is accompanied by an exothermic effect, with a maximum of 335.6 °C. The recorded mass loss is 12.60%. The last recorded mass loss, 10.67%, takes place after 440 °C (Figure 5). It can be attributed to the decomposition of the Na_2_CO_3_ formed during the previous processes, the strong exothermic effect from 539.8 °C being due to the Na_2_O reaction with the Al_2_O_3_ crucible. The main component of the evolved gases, after 300 °C, is the CO_2_, with some traces of H_2_O and C-H fragments observable under 440 °C.

The 5% BC-Nutmeg sample presents a mass loss of 10.17% up to 160 °C. This process can be attributed to the loss of water molecules, physically adsorbed by the cellulosic structure, and to the volatile components from the essential oil. An endothermic effect with a minimum of 98.3 °C accompanies the process. In the second process, between 160–300 °C, the cellulosic fibers start to decompose, and the essential oil components are eliminated. The recorded mass loss value is 45.97%. An endothermic effect with a minimum of 242.2 °C accompanies this process. The FTIR spectra recorded for the evolved gases indicate the presence of principally water and carbon dioxide molecules, but also some traces of carbon monoxide and hydrocarbon fragments from the essential oil components and cellulose decomposition. The carbonaceous residue burns off at the temperature interval of 300–440 °C; the process is accompanied by multiple exothermic effects, with maxima at 326.7, 378.9, and 406.3 °C. The recorded mass loss is 37.15%. The last recorded mass loss, 7.95%, occurred over 440 °C. It can be attributed to the decomposition of the Na_2_CO_3_ formed during the previous processes, the strong exothermic effect from 542.3 °C being due to the Na_2_O reaction with the Al_2_O_3_ crucible. The main component of the evolved gases, over 300 °C, is the CO_2_, with some traces of H_2_O and C-H fragments observable under 440 °C. Principal data from thermal analysis of BC and BC-Nutmeg samples are presented in Table 1.

**Figure 6:**
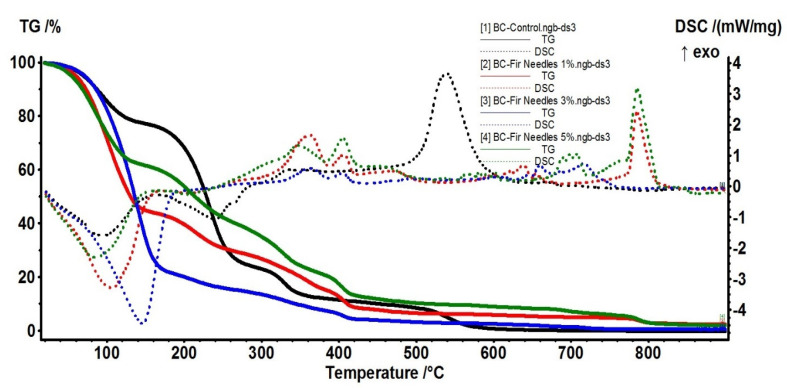
TG-DSC curves for BC-Control, BC-Fir needle 1%, BC-Fir needle 3%, BC-Fir needle 5%, and Fir needle essential oil.

In Figure 6, the 5% BC-Fir needle sample shows a mass loss of 39.27% up to 160 °C. This process can be attributed to the loss of water molecules, physically adsorbed by the cellulosic structure, and to the volatile components from the essential oil. An endothermic effect, with a minimum of 84.1 °C, accompanies the process. The peak of the endothermic effect occurs at lower temperatures, and the mass loss is higher than for the simple cellulose, indicating that some volatile components from the essential oil are indeed eliminated.

**Figure 7:**
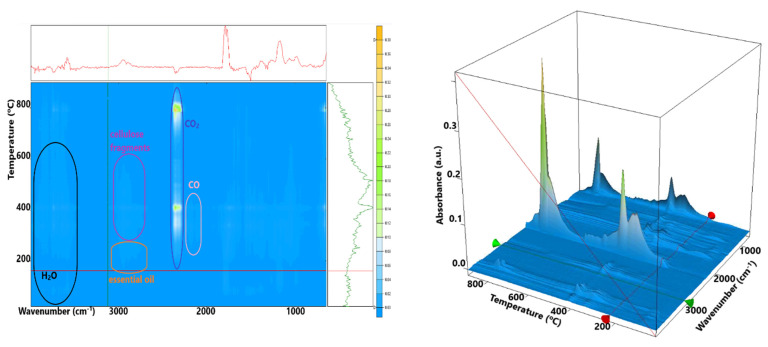
The 2D projection of the FTIR spectra vs. temperature and the 3D diagram of FTIR vs. temperature for the evolved gases for the 5% Fir needle sample.

In the second process, between 160–300 °C, the cellulosic fibers start to decompose, and as the FTIR spectra of evolved gases indicate, the essential oil components are eliminated (Figure 7). The recorded mass loss value is 25.67%. A weak endothermic effect with a minimum of 202.5 °C accompanies this process. The FTIR spectra recorded for the evolved gases indicate the presence of principally water and carbon dioxide molecules, but also some traces of carbon monoxide and hydrocarbons fragments from essential oil components and cellulose decomposition.

The carbonaceous residue is burned off at the temperature interval of 300–440 °C; the process is accompanied by multiple exothermic effects, with maxima at 346.8 and 405.3 °C. The recorded mass loss is 22.86%. The last recorded mass loss, 10.16%, takes place over 440 °C. It can be attributed to the decomposition of the Na_2_CO_3_ formed during the previous processes, the strong exothermic effect from 785.2 °C being due to the Na_2_O reaction with the Al_2_O_3_ crucible. The main component of the evolved gases, after 300 °C, is CO_2_, with some traces of H_2_O and C-H fragments observable under 440 °C. Principal data from thermal analysis of BC and BC-Fir needle samples are presented in Table 2.

### 3.4. Kinetics of Water Absorption

The water absorption capacity plays a significant role in assessing the hydration capacity of BC membranes. An important feature of these membranes is directly associated with the hydrophilicity of BC, which helps absorb the wound exudate, ensures rapid healing, and makes the membrane removal painless. BC absorption data demonstrated the a superior ability to absorb alkaline fluid than acidic fluid [48]. Several studies have investigated the relationship between wound pH and chronic wound healing [15].

**Figure 8:**
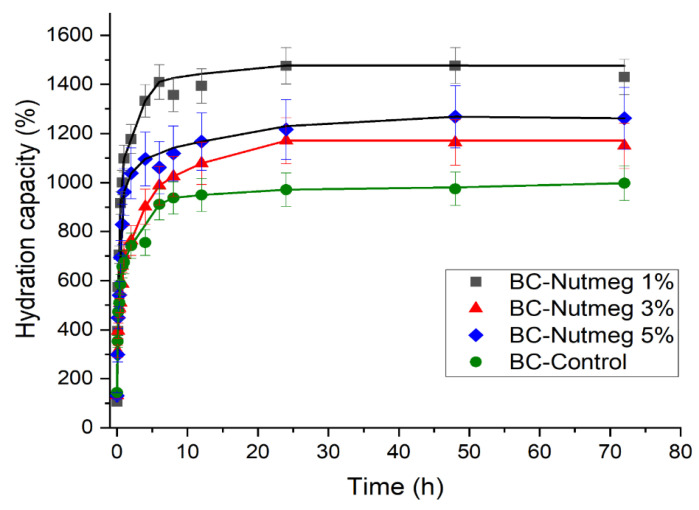
Absorption kinetics of samples BC-Control, BC-Nutmeg 1%, BC-Nutmeg 3% and BC-Nutmeg 5%.

For the BC-Nutmeg samples compared to the simple BC sample (Figure 8), there is an increased absorption ability, due to the release of the essential oil and the high absorption capacity of the membrane. It can be seen that the highest degree of absorption is obtained for the BC-Nutmeg sample of 1%, followed by the 5% BC-Nutmeg sample and then the sample with a 3% emulsion concentration of nutmeg essential oil. The water absorption is strongly improved with the incorporation of the nutmeg essential oil into the BC membrane, even though the water absorption is not proportional to the amount of nutmeg oil emulsion added.

**Figure 9:**
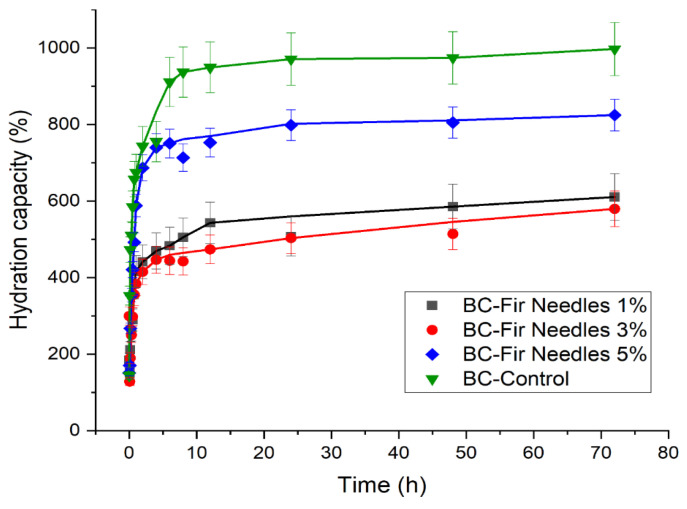
Absorption kinetics of the BC-Control, BC-Fir needle 1%, BC-Fir needle 3%, and BC-Fir needle 5% samples.

Figure 9 shows the evolution of the water absorption of samples loaded with fir needle essential oil emulsion. In these cases, there is a rapid increase in the first 30 min, stabilizing after about 12 h. This fast absorption rate in the first few minutes results in rapid removal of excess exudate from the wound and its protection from pathogens. For BC fir needle oil loaded with 1% and 3% fir needle essential oil, the maximum absorption capacity of the emulsions is approximately 550%. For the BC fir needle 5% sample, this is approximately 750%, and for the pure BC sample, the maximum absorption capacity is approximately 1000%.

It can be seen that the addition of nutmeg essential oil leads to improved water absorption, whereas fir needle essential oil leads to a reduced water absorption rate. These results can be explained by the composition of these essential oils, which when absorbed into the BC fibers, probably induce a more hydrophobic or hydrophilic behavior. In both cases, when the essential oil content increases to 5%, an abnormal evolution is observed, which can likely be explained by the association of the essential oil molecules with each other and not with the BC fibers.

### 3.5. Gas Chromatographic—Mass Spectrometric Evaluation

**Figure 10:**
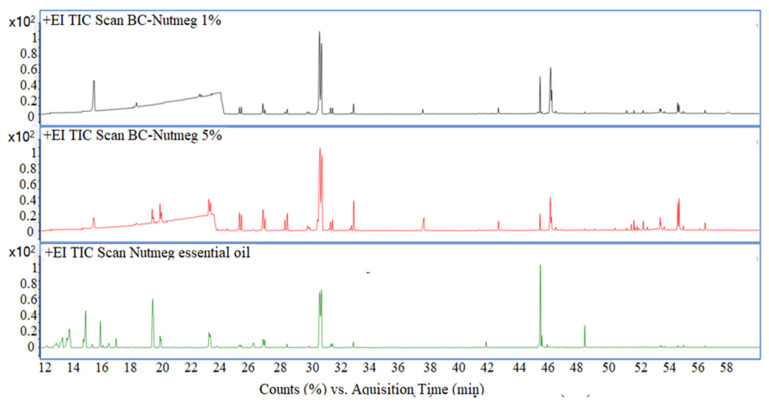
Total ion chromatogram of BC-Nutmeg 1%, BC-Nutmeg 5%, and nutmeg essential oil.

**Figure 11:**
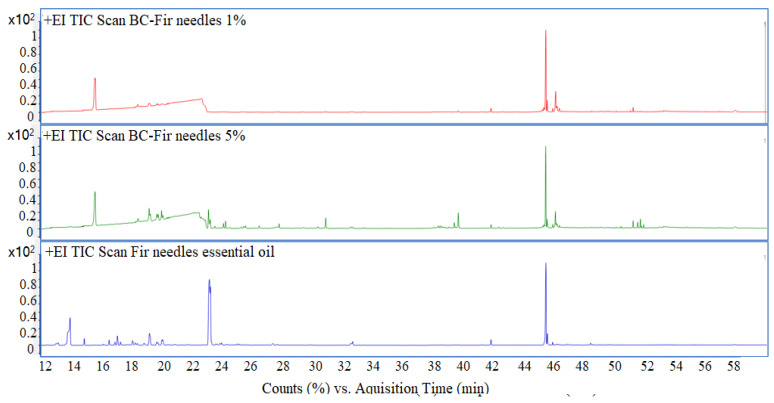
Total ion chromatogram of BC-Fir needle 1%, BC- Fir needle 5%, and fir needle essential oil.

GC-MS data of the BC samples loaded with 1 and 5% Nutmeg and Fir Needle Eos, respectively, are presented in Figure 10 and Figure 11, proving the existence of the main peaks of these essential oils.

A series of compounds were identified for the obtained materials, which are presented in Table 3. A number of volatile compounds characteristic of the essential oils were identified in the BC samples, demonstrating the interactions of the essential oils with the BC material. At RT = ~21 min, the peak of glycerol from the BC material is observed.

Corroborating the data from the total ion chromatograms (Figure 10 and Figure 11) and the assignments of the peaks with the components, it can see that several peaks decreased considerably as area (content) decreased, such as, for instance Bornyl Acetate (for the samples loaded with fir needle EO) and terpineol (for the samples loaded with nutmeg EO). Also, some of the most volatile compounds (which are found in low concentrations) are mostly lost during the handling.

### 3.6. Biocompatibility

The MTT (3-(4,5-dimethylthiazol-2-yl)-2,5-diphenyltetrazolium bromide) addresses the cell metabolic activity. NAD(P)H-dependent cellular oxidoreductase enzymes are capable of reducing the MTT tetrazolium dye and, in certain conditions, can reflect the number of viable cells present.

**Figure 12:**
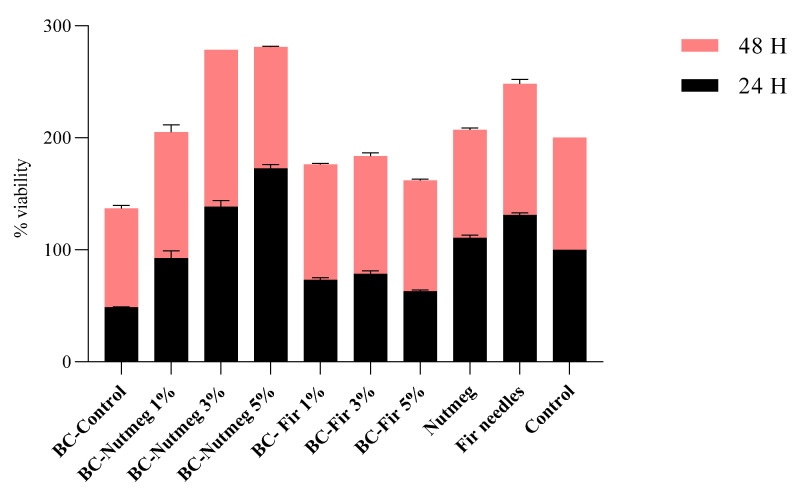
The viability rate of L929 cells after 24 h and 48 h exposure to samples BC-Control, BC-Nutmeg 1%, BC-Nutmeg 3%, BC-Nutmeg 5%, BC-Fir needle 1%, BC-Fir needle 3%, BC-Fir needle 5%, and Nutmeg oil and Fir needle oil controls; Control—L929 cells in culture media (*p*-value = 0.003).

After 24 h of exposure to the samples, the cell viability was below 50% for BC-Control, and under 70% for BC-Fir needle 1%, BC-Fir needle 3%, and BC-Fir needle 5%, as can be seen in Figure 12. This fact may be attributed to the initial shock of the cells, a stress response to new substances introduced into the medium, or due to the high release rate of the incorporated actives [49]. As for samples BC-Nutmeg 1%, BC-Nutmeg 3%, BC-Nutmeg 5%, and Nutmeg control, the viability rates were over 80%, even at the 24 h mark. After 48 h of exposure, all samples presented high rates—over 85%—of viability (Figure 12). The *p*-value was <0.05, and it is considered that the data results indicate good biocompatibility and efficient cellular proliferation.

Increased viability percentages were obtained after 48 h of exposure to tested compounds compared to the 24 h results. These results provide good exclusion criteria for some samples, indicating the need for the optimization of controlled release of the EOs.

Several studies report the usage of EO in wound care products due to their wound healing and anti-inflammatory properties [50,51]. More specifically, fir needle and nutmeg oils have been reported for promoting wound closure and microbial management effects until systemic treatment with antibiotics is administered [52,53,54]. Keeping this in mind, the topical and transdermal application of EO active ingredients must be safe and non-toxic (without causing irritation), as well as structurally protected, without affecting their intrinsic ability to permeate lipophilic biological membranes or to interact with various receptors in the body [55].

Results obtained for the biocompatibility assay indicate the impact of the BC network on cellular proliferation, migration, and initial viability markers at 24 h after the initial contact. Due to its compact structure, characterized by decreased hydrophilicity, notable roughness, and stiffness, cellular propagation were slowed down as compared to those noted in the control. This initial impact effect can also be observed for the 1, 3, and 5% fir needle samples. It seems that nutmeg oil acts as a permeant, decreasing fiber stiffness (fiber relaxation) and promoting cellular proliferation. Since the relaxation phenomenon and swelling behaviors of cellulose fibers (especially regenerated ones) tend to be affected by water [56,57], the incorporation of EOs can aid in the biotechnological improvement of this shortcoming.

### 3.7. Antibacterial Assays

Wounds are susceptible for infections, and this is why the antimicrobial activity of the wound dressing is beneficial, especially if this activity is not assured by the use of antibiotics or other agents able to induce resistance. The occurrence of drug-resistant microorganisms, like *S. aureus* and *E. coli,* due to the uncontrolled usage of common antibiotics and synthetic antimicrobials, is a challenge for researchers who are working to find alternative and effective ways to treat such diseases. EOs and their multi-target components are alternative antimicrobial compounds that reduce the possibility of developing microbial resistance. EOs affect the permeability of the bacterial cell wall and cytoplasmic membrane, followed by ion loss, reduced membrane potential, proton pump collapse, and depletion of the ATP reservoir [58].

**Figure 13:**
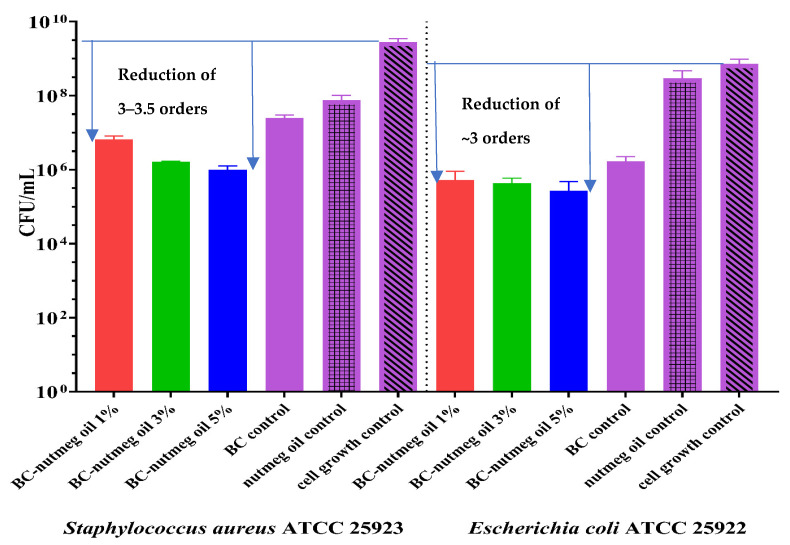
Graphical representation of CFU/mL values of nutmeg EO-based BC samples against *S. aureus ATCC 25923* and *E. coli ATCC 25922* to assess their bacterial ability to adhere to sample areas consisting of BC-Control, BC-Nutmeg 1%, BC-Nutmeg 3% and BC-Nutmeg 5%. The antibacterial effect of the membranes against these strains was compared using one-way ANOVA and Dunnett’s multiple comparisons tests. The *p*-value is <0.0001 and is considered statistically significant.

Figure 13 shows that CFU/mL values decrease for all the materials containing nutmeg essential oil compared to plain BC, particularly regarding cell growth control. Moreover, it is necessary to mention that synergy is obtained when nutmeg essential oil is adsorbed onto the BC support. Consequently, the CFU/mL values for the BC-Nutmeg samples at all three concentrations exhibit a stronger activity than does pure nutmeg essential oil. In the case of the sample loaded with 5% nutmeg oil, a decrease of approximately two logarithmic units can be observed in both strains, resulting in the capacity to inhibit the adherence of bacteria to the surface of the materials. The other concentrations used in making the materials, 1% and 3%, respectively, showed a slightly lower ability to inhibit the adhesion of microorganisms.

EOs are some of the most promising and inexpensive antimicrobials, not only because of their activity, but also due to their synergism when combined with conventional antibiotics and synthetic drugs, reducing the dose of the latter.

**Figure 14:**
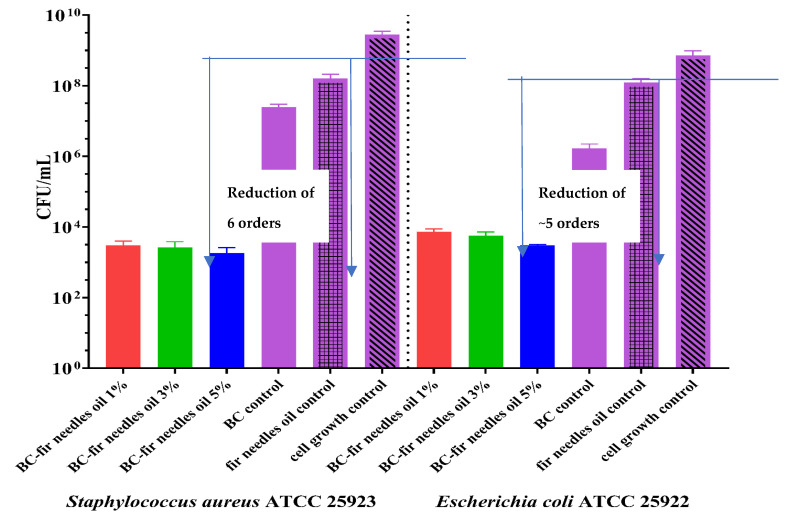
Graphical representation of CFU/mL values of fir needles EO-based BC samples against *S. aureus ATCC 25923* and *E. coli ATCC 25922* to assess their bacterial ability to adhere to sample areas of BC-Control, BC-Fir needles 1%, BC-Fir needle 3%, and BC-Fir needle 5%. The antibacterial effect of the membranes against the strains was compared using one-way ANOVA and Dunnett’s multiple comparisons tests. The results were considered statistically significant (*p* < 0.0001).

According to literature studies, fir essential oils and essential conifer extracts exhibit antibacterial properties, antioxidant activity, and anticancer properties [23,31,59]. By performing antibacterial tests on membranes loaded with fir needle essential oil, we demonstrated the ability of these membranes to inhibit bacterial adhesion to the substrate. As can be seen in Figure 14, the presence of fir needle essential oil managed to reduce the adhesion of bacterial cells on the membrane surface by about 4 logarithmic units, for samples with 5% oil concentration, and about 3 logarithmic units, for samples with 1% and 3% oil concentrates. Thus, it can be seen that these membranes can inhibit the adherence of *S. aureus* and *E. coli* bacterial strains, and they could be good candidates for the prevention and treatment of *S. aureus* and *E. coli* infections associated with skin wounds.

Both types of loaded samples maintain their antimicrobial activity, even if some more volatile compounds are partially or totally evaporated. The samples loaded with fir needle EO exhibit much higher antimicrobial activity (the reduction of the CFU/mL being of 5–6 orders, which means a reduction of 99.999–99.9999%), but have a lower water absorption capacity, so its use is especially recommended for non-suppurative or medium-suppurative wounds, while the BC membrane loaded with nutmeg can be used for the suppurative wounds, even if the antimicrobial activity in lower (at least compared with the previous activity), but it is still adequate, the reduction of the CFU/mL being ~3 orders (comparing with the cell growth control).

The good antimicrobial activity in no way compromises the viability rate of the L929 cells, which means that these compositions are safe for use in wound dressing applications.

## 4. Conclusions

The present study aims to develop membranes with antimicrobial properties provided by two essential oils, nutmeg and fir needle, loaded into their structure. Natural essential oils were chosen in order to find alternatives to the use of antibiotics (at least for preventive purposed) because their overuse can generate resistance. The GC-MS data prove the presence of some natural compounds that make up the composition of the essential oils studied in this article. Some of these compounds are responsible for the antimicrobial activity of these oils, as confirmed by the literature, and these components can be observed and released from the membranes, as proved by the GC-MS analyses. The obtained results demonstrated the anti-adherent properties of these membranes used as a preventive/treatment method for skin infections associated with *S. aureus* ATCC 25923 and *E. coli* ATCC 25922. Microscopic analyses show the porous structure of these cellulose-based membranes. Together with the hydration capacity analysis, it can be concluded that these membranes have a high absorption capacity suitable for treating infected wounds, especially highly suppurative ones. The selection of the wound dressings (the type of essential oil) can be associated with the required absorption capacity. If the wound is highly suppurative, the nutmeg EO can be used because the absorption capacity reached ~1500% for the sample loaded with 1% nutmeg, while the CFU/mL is reduced ~3 orders of magnitude. In the case of fir needle EO-based BC, the hydration is well below the hydration capacity of the BC, but the CFU/mL is reduced by almost 6 orders of magnitude (reported for cell growth control). Further works will be devoted to evaluating the symbiotic use of the two Eos, as well as their combination with antibiotics in standardized, but also clinical, bacterial strains (including multidrug-resistant bacterial strains).

## Figures and Tables

**Table 1:** Main effects and associated mass changes recorded for the BC, and BC-Nutmeg 1, 3, and 5%.

Sample	Temperature (°C)	Mass Loss (%)
**BC-Control**	160	23.51
	160–300	53.51
	300–440	12.60
	>440	10.67
**BC-Nutmeg 1%**	160	23.63
	160–300	50.71
	300–440	19.21
	>440	6.34
**BC-Nutmeg 3%**	160	19.39
	160–300	44.74
	300–440	28.33
	>440	7.23
**BC-Nutmeg 5%**	160	10.17
	160–300	45.97
	300–440	37.15
	>440	7.95

**Table 2:** Main thermal effect and mass changes associated with the BC and BC-FN 1, 3, and 5%.

Sample	Temperature (°C)	Mass Loss (%)
**BC-Control**	160	23.51
	160–300	53.51
	300–440	12.60
	>440	10.67
**BC-Fir needle 1%**	160	55.96
	160–300	17.26
	300–440	18.73
	>440	5.70
**BC-Fir needle 3%**	160	73.24
	160–300	13.22
	300–440	9.49
	>440	3.49
**BC-Fir needle 5%**	160	39.27
	160–300	25.67
	300–440	22.86
	>440	10.16

**Table 3:** GC-MS-based identification of the main components of the BC-EO.

Samples	Compounds	Retention Time (RT)
**BC/Fir needle essential oil**	D-Limonene	13.698
	Pinene epoxide	16.299
	Fenchol	17.058
	Sabinol	17.849
	Endo-Borneol	18.973
	Bornyl acetate	22.936
	D-Verbenone	23.654
	Longifolene	27.191
	Caryophyllene oxide	32.341
	2-Methylisoborneol	30.666
	Palmitic acid, methyl ester	41.641
	Oleic acid, methyl ester	45.282
	Methyl stearate	45.730
**BC/Nutmeg essential oil**	γ-Terpinene	14.794
	Terpinolene	15.782
	Terpineol	19.288
	Safrole	23.065
	Eugenol	25.218
	Methyleugenol	26.685
	trans-Isoeugenol	28.163
	Myristicin	30.607
	Elemicin	31.346
	Methoxyeugenol	32.772
	Ricinoleic acid methyl ester	48.261
	Licarin A	53.308
	6-Methoxyeugenyl isovalerate	54.577

## Data Availability

Data is contained within the article.

## Cost Action

CA18132—Functional Glyconanomaterials for the Development of Diagnostics and Targeted Therapeutic Probes (GLYCONanoPROBES).
